# Novel bisphosphonate-based cathepsin K-triggered compound targets the enthesis without impairing soft tissue-to-bone healing

**DOI:** 10.3389/fbioe.2024.1308161

**Published:** 2024-02-16

**Authors:** Brendan Y. Shi, Varun Sriram, Shannon Y. Wu, Dave Huang, Alexis Cheney, Melodie F. Metzger, Oskar Sundberg, Karen M. Lyons, Charles E. McKenna, Ichiro Nishimura, Thomas J. Kremen

**Affiliations:** ^1^ Department of Orthopaedic Surgery, David Geffen School of Medicine, University of California at Los Angeles, Los Angeles, CA, United States; ^2^ Department of Orthopaedic Surgery, Cedars-Sinai Medical Center, Los Angeles, CA, United States; ^3^ Department of Chemistry, University of Southern California, Los Angeles, CA, United States; ^4^ Department of Molecular, Cellular, and Developmental Biology, University of California at Los Angeles, Los Angeles, CA, United States; ^5^ Weintraub Center for Reconstructive Biotechnology, School of Dentistry, University of California at Los Angeles, Los Angeles, CA, United States

**Keywords:** enthesis, biomechanics, growth factor delivery, rotator cuff repair, targeted delivery

## Abstract

**Background:** Osteoadsorptive fluorogenic sentinel 3 (OFS-3) is a recently described compound that contains a bone-targeting bisphosphonate (BP) and cathepsin K (Ctsk)-triggered fluorescence signal. A prior study in a murine Achilles repair model demonstrated its effectiveness at targeting the site of tendon-to-bone repair, but the intrinsic effect of this novel bisphosphonate chaperone on tendon-to-bone healing has not been previously explored. We hypothesized that application of this bisphosphonate-fluorophore cargo conjugate would not affect the biomechanical properties or histologic appearance of tendon-bone repairs.

**Materials and Methods:** Right hindlimb Achilles tendon-to-bone repair was performed on 12-week old male mice. Animals were divided into 2 groups of 18 each: 1) Achilles repair with OFS-3 applied directly to the repair site prior to closure, and 2) Achilles repair with saline applied prior to closure. Repaired hindlimbs from 12 animals per group were harvested at 6 weeks for biomechanical analysis with a custom 3D-printed jig. At 4 and 6 weeks, repaired hindlimbs from the remaining animals were assessed histologically using H&E, immunohistochemistry (IHC) staining for the presence of Ctsk, and second harmonic generation (SHG) imaging to evaluate collagen fibers.

**Results:** At 6 weeks, there was no significant difference in failure load, stiffness, toughness, or displacement to failure between repaired hindlimbs that received OFS-3 *versus* saline. There was no difference in tissue healing on H&E or Ctsk staining on immunohistochemistry between animals that received OFS-3 *versus* saline. Finally, second harmonic generation imaging demonstrated no difference in collagen fiber parameters between the two groups.

**Conclusion:** OFS-3 did not significantly affect the biomechanical properties or histologic appearance of murine Achilles tendon-to-bone repairs. This study demonstrates that OFS-3 can target the site of tendon-to-bone repair without causing intrinsic negative effects on healing. Further development of this drug delivery platform to target growth factors to the site of tendon-bone repair is warranted.

## 1 Introduction

Roughly half of the 32 million musculoskeletal injuries that occur annually in the United States affect tendons or ligaments (Butler et al., 2004). Rotator cuff disease, specifically, has been shown to have a prevalence of 7%–10% in patients younger than 30 and a prevalence upwards of 30% in patients 60 and older (Teunis et al., 2014). As the elderly population continues to expand, the number of rotator cuff repairs performed per year continues to rise (Colvin et al., 2012; Yanik et al., 2021). Unfortunately, the reported rate of recurrent or persistent tears after repair ranges from 11% to 20% (Gerber et al., 2000; Slabaugh et al., 2010), with some reports as high as 94% (Galatz et al., 2004). Despite significant advances in arthroscopic technology and increased use of biomechanically superior transosseous equivalent techniques, recent studies have continued to report similar recurrent tear rates (Kim and Kim, 2016).

One major reason for rotator cuff repair failure is the inability of current surgical techniques to restore the tissue structure and biomechanical properties found at the native enthesis (Genin et al., 2009; Kanazawa et al., 2016; Derwin et al., 2018). Instead, rotator cuff tendons repaired to bone form poorly organized fibrovascular scar tissue that are compositionally deficient, lacking adequate organization of Type 1 collagen and fibrocartilage among other elements (Murray et al., 2007). Although growth factors including bone morphogenic proteins (BMPs) have been shown to improve the organization, fiber orientation, and biomechanical strength of tendon-to-bone repairs (Murray et al., 2007; Rodeo et al., 2007; Kovacevic et al., 2011; Pauly et al., 2012), the lack of targeted delivery options remains a significant barrier to wide spread use of growth factor-based therapies.

During surgical tendon-to-bone repair, local bone is mechanically disrupted, exposing hydroxyapatite (HAP) mineral matrix, and undergoes remodeling with localized osteoclastic bone resorption (Cole et al., 2016). Given their affinity for HAP, bisphosphonates (BP) are a logical option for targeting the repair site (Le et al., 2014). Osteoadsorptive Fluorogenic Sentinel 3 (OFS-3) is a recently described molecule (Richard et al., 2021) that contains a BP molecule conjugated to fluorochrome F) and quencher Q) molecules. The quencher, which inhibits emission of fluorescence when in close proximity to the fluorochrome, is linked to the BP-F parent molecule via a peptide sequence that is sensitive to the actions of the osteoclast-derived protease, cathepsin K (Ctsk). Thus, not only is OFS-3 targeted to hydroxyapatite minerals in bone, like all BPs, but release of OFS-3’s coupled moieties is limited to areas with high osteoclast (OC) activity such as those found at the surgical site after tendon-to-bone repair (Kremen et al., 2023). Using a murine Achilles repair model, we recently demonstrated that OFS-3 can effectively target the site of tendon-to-bone repair whether applied locally at the time of surgery or administered via systemic injection after surgery (Kremen et al., 2023).

However, while OFS-3 represents a useful tactic for bringing coupled moieties to the site of bone remodeling, BPs potential inhibition of osteoclast activity (Hughes et al., 1995; Drake and Cremers, 2010) may have unintended effects separate from their role as a delivery mechanism. Prior studies have demonstrated that BPs can impair fracture healing (Savaridas et al., 2013), may reduce bone resorption at the tendon-bone interface (Thomopoulos et al., 2007), and may reduce the strength and stiffness of the tendon-bone interface (Hjorthaug et al., 2018). OFS-3 utilizes a modified pamidronate BP molecule designed to have significantly decreased biological activity due to changing the side chain amino group to an *N*-substituted amide. This alteration is expected to result in significantly lowered farnesyl pyrophosphate synthase (FPPS) activity (Tsoumpra et al., 2015; Sung et al., 2020; Okawa et al., 2022) both due to the amine/amide modification and the introduction of a sterically bulky amido substituent. However, the effect of OFS-3 on tendon-to-bone healing has not been explored previously.

This study aimed to evaluate the feasibility of using OFS-3 to target the site of tendon-bone repair without impairing the strength of repair or the biology of healing using a murine model of tendon-bone healing. We hypothesized that OFS-3 would not affect the biomechanical properties, histologic features or degree of Ctsk activity in tendon-to-bone repairs.

## 2 Materials and Methods

### 2.1 Molecule synthesis

OFS-3 was synthesized in the laboratory of Charles McKenna, PhD (Richard et al., 2021). Once obtained, it was suspended in sterile phosphate buffered saline at a pH of 7.4. As described by Richard et al. (Richard et al., 2021), OFS-3 contains a modified pamidronate (BP) molecule conjugated to a sulfo-cyanine 5 fluorochrome and BlackBerry Quencher 650 (Berry & Associates, Dexter, MI) which quenches any external fluorescence emitted from the sulfo-cyanine 5. However, since the quencher is conjugated to the BP molecule via a peptide with the Ctsk-sensitive sequence GHPGGPQG, in the presence of Ctsk, the peptide sequence is cleaved and the quencher is released, allowing fluorescence to be observed externally.

### 2.2 Surgical procedures

After approval by our institution’s Animal Research Committee, thirty-six 12-week-old male C57BL/6 mice (Charles River Laboratories, Wilmington, MA) underwent right hindlimb surgery for either Achilles tendon-to-bone repair or sham surgery as previously described (Kremen et al., 2023). In brief, the skin on the lateral side of the right Achilles tendon was first incised sharply to expose the tendon. For animals receiving Achilles repair, 5–0 nylon suture was used to place a transverse stitch through the Achilles tendon and the posterior calcaneus. After capturing these tissues, the Achilles tendon was sharply transected at its calcaneus attachment and the bony footprint was decorticated with a dental burr. The tendon was then repaired to bone using the previously placed 5–0 nylon suture.

All animal procedures were performed under isoflurane anesthesia using standard aseptic technique. Animals were administered long-acting buprenorphine once immediately prior to the procedure and once more at 72 h post-operatively. After surgery, animals were monitored daily for signs of stress and to ensure adequate food intake and hydration.

### 2.3 Biomechanical assessment

Our biomechanical assessment experiments included 24 mice randomized into two groups of 12 animals. The Achilles repair treated with OFS-3 (AR + OFS3) group underwent Achilles tendon-to-bone repair followed by application of 5 μL of 1,000 nM OFS-3 via micro-pipet directly onto the tendon-bone junction at the repair site prior to skin closure. The Achilles repair (AR) group (Saline/Control group) underwent Achilles tendon-to-bone repair followed by application of 5 μL of sterile saline via micro-pipet directly onto the repaired bone-tendon junction prior to skin closure.

Six weeks after surgery, all animals were euthanized. Repaired hindlimbs were harvested and frozen. Specimens were taken out to thaw 1 h prior to testing. Once thawed, the tibia and fibula were removed and the soft tissue was dissected away, leaving the hindfoot and repaired Achilles tendon. To minimize disruption of the fibrotic scar formation at the repair site, the surgically placed suture was not removed.

**FIGURE 1:**
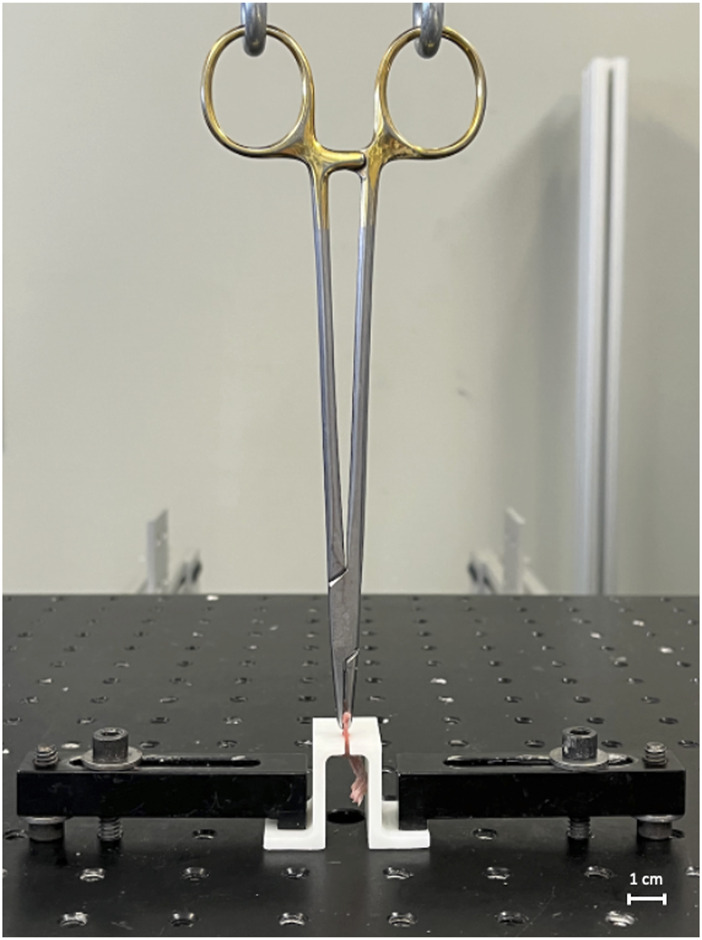
Biomechanical testing apparatus. The apparatus consists of a hydraulic testing machine and custom 3D-printed jig.

The proximal end of the Achilles tendon was captured 3 mm (measured using digital calipers) from the tendon-bone junction using a needle driver that was attached to the actuator of a mechanical testing system (370.02 Bionix, MTS Systems Corp., Eden Prairie, MN). The calcaneus was secured to the frame of the testing machine via a custom 3D printed jig (Figure 1). Mounted specimens were loaded at a rate of 0.15 mm/s until failure while tensile force and displacement were continually recorded. While preconditioning is typically recommended when analyzing viscoelastic materials like tendons and ligaments, we deviated from these standard procedures to ensure consistency and specificity in our measurements as preconditioning could potentially alter the natural mechanical responses of this complex interface. This methodology is consistent with prior research studies analyzing the bone-tendon interface (Bell et al., 2015; Cong et al., 2018). Maximum failure load N), maximum displacement (mm), stiffness (N/mm), and toughness (Nmm) were determined from the resultant force-displacement curve.

Prior to testing repaired specimens, pilot testing on identically prepared unrepaired murine hindlimbs was performed, demonstrating that the described clamping system and loading protocol had high reproducibility and yielded low variance in all recorded outcomes. To evaluate the biomechanical contribution of the suture material itself, 5–0 nylon suture loops were also tested to failure with the same loading protocol.

### 2.4 Histologic analysis

Our histologic analysis included 12 animals randomized into two groups of 6. Similar to the biomechanical experiments, the AR + OFS3 group underwent Achilles repair followed by direct OFS-3 application, whereas the AR group (Saline/Control) underwent Achilles repair followed by saline application. Hindlimbs were harvested from 3 animals per group at 4 weeks and 6 weeks after surgery.

Each hindlimb sample was fixed in 4% paraformaldehyde for 3 days before being decalcified in formic acid for 2 days and embedded in paraffin. Sagittal sections 5 µm thick were generated with a microtome. Representative sections from 3 separate regions of the tendon-bone junction (medial, mid-sagittal and lateral) per mouse were selected for histologic analysis and underwent staining with hematoxylin and eosin (H&E). Sections immediately adjacent to the 3 regions selected for H&E underwent immunohistochemistry (IHC) staining for the presence of Ctsk using cathepsin K antibodies (Abcam, Cat: ab19027) at a dilution of 1:100 using standard protocols used in the translational pathology core at our institution as previously described (Littlewood-Evans et al., 1997). DAB (3, 3’ diaminobenzidine) methodology was then used to visualize the degree of cathepsin K antibodies (Binch et al., 2020). Finally, representative sections from the mid-sagittal region of the tendon-bone junction were selected for second harmonic generation (SHG) imaging to assess collagen fiber orientation and size.

After H&E and IHC staining, images were acquired with the Keyence BZ-X800 microscope using a ×10 objective lens. The H&E-stained sagittal sections from both OFS-3 treated animals and saline control animals (3 sections per mouse) were then compared via qualitative assessment of the anterior-to-posterior size of the reparative tissue. In addition, the degree of Ctsk staining was compared. Qualitative assessments were performed by two independent examiners trained in murine musculoskeletal histology and blinded to treatment group.

Next, the ratio between osteoclast (OC) surface area and total bone surface area was quantified using previously described methods (Lemmon et al., 2018). Brightfield images of stained tissue sections from 3 animals in each treatment group at 6 weeks after tendon-bone repair were acquired using the Leica SP8 MP-DIVE two-photon microscope (Leica Camera, Wetzlar, Germany). Images were collected using a 40x (NA = 1.25) oil immersion objective lens and 12-bit Leica DFC420 camera (pixel size of 0.162 microns) with a 300 m exposure time. A commercial AI-based image analysis software (Aivia 13.0, Leica Microsystems, Wetzlar, Germany) was used to quantify the OC/bone surface area ratio at the site of bone-tendon repair within each image. The pixel-classifier tool was used to generate OC and bone area confidence maps, first on relatively small regions within a single image. The classifier was then saved and applied on the full region of interest (ROI) of the training image, followed by unseen images captured under similar conditions. In all training scenarios, sub-optimally labelled regions were identified and corrected in an iterative fashion until satisfactory results were observed and agreed upon by 3 observers. As staining conditions varied, additional training regions taken from 5 different images were added to the training set. The AIVIA built-in ‘Recipe’ called ‘Cell Count’ was then used on the resultant masks to segment regions of high confidence and calculate the total area of the segmented objects for each mask.

SHG imaging of collagen fibers were obtained with the Leica SP8 microscope using a method adapted from Schlegel et al. (Schlegel et al., 2023). For 10x imaging, unstained sections were imaged using an excitation wavelength of 830 nm at ∼580 mW (30% of maximum laser power) with backwards SHG signal collected in reflectance mode from 405 to 425 nm. Tile scans were taken using a ×10 dry objective lens and qualitatively assessed for the location of regions of interest. Once the regions of interest were identified, images of these regions were obtained using a ×40 water immersion objective lens (NA = 1.1) with an excitation wavelength of 830 nm at 400 mW (20% of maximum laser power). Tile scans were taken and cropped into regions of interest of ∼150 × 150 microns. Three regions of interest per mouse in each treatment group were used for the analysis.

Collagen fiber alignment was assessed using the open-source, MATLAB-based software tools CurveAlign version 5.0 (University of Wisconsin-Madison, Madison, WI) and CT-FIRE version 3.0 (University of Wisconsin-Madison, Madison, WI) for curvelet transform-based fibrillar collagen quantification (Liu et al., 2017; Liu et al., 2020). Absolute angles measured between the fiber and horizontal axis (ranging from 0 to 180°) are converted to circular angles, and their mean value is divided by 2 to determine orientation. The length of the sum of orientation vectors divided by the total number of angles is then used as the alignment metric, with values ranging from 0 (not aligned) to 1 (perfectly aligned). Collagen fiber alignment values consisted of the alignment of each collagen fiber relative to its neighbors within 2 pixels, 4 pixels, 8 pixels, and 16 pixesl. The mean alignment value used for analysis was defined as the mean of each of these pixel distances for each fiber.

### 2.5 Statistical analysis

Statistical analysis of normally distributed histologic data including the collagen fiber assessments were performed by comparing the means from each treatment group using a random effects (mixed) analysis of variance model where region within each mouse was a random effect as well as within region measurement error (replicate error). OC/bone surface area ratio distribution did not follow the normal distribution and, thus, the *p* values were computed using the non-parametric Kruskal–Wallis one-way analysis of variance.

Biomechanical outcomes between groups were compared with one-way ANOVA and Tukey’s *post hoc* test, with alpha set at 0.05. Stata 12 Software (StataCorp LLC, College Station, TX) was used for statistical analyses.

A prospective power analysis was performed using data from pilot testing on unrepaired murine hindlimbs (mean failure load 11N, standard deviation 1.6). Twelve animals per group were deemed necessary to detect a 2N difference in load to failure with a power of 0.80 (G*Power Version 3.1.9, Düsseldorf, Germany).

## 3 Results

### 3.1 Biomechanical assessment

**FIGURE 2:**
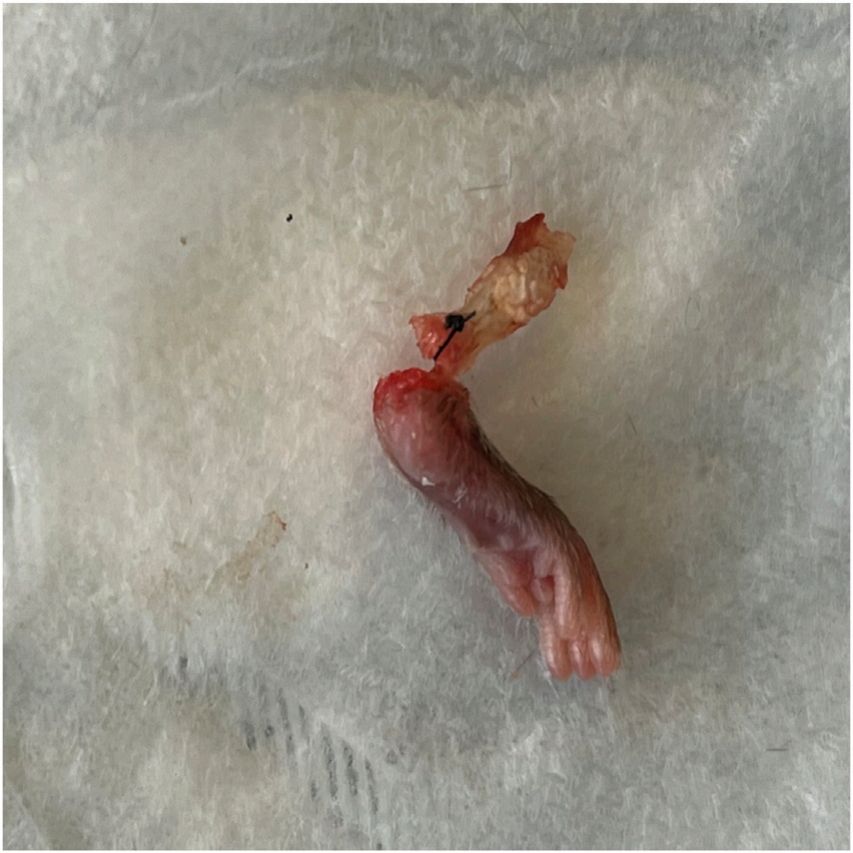
Representative specimen after load to failure. All specimens failed at the junction of the tendon/reparative tissue and bone.

There was no significant difference in ultimate failure load, maximum displacement, stiffness, or toughness between repaired hindlimbs that received local OFS-3 and those that received saline (Table 1). The mean failure load was 17.4 N in animals that received local OFS-3 after repair and 16.0 N in saline control animals (*p* = 0.440). Mean maximum displacement values for the Achilles repair (AR), Achilles repair plus OFS3 (AR + OFS3), and uninjured groups were 1.9 mm, 2.2 mm, and 1.9 mm, respectively. All tested specimens failed at the tendon/reparative tissue-bone junction (Figure 2).

**TABLE 1:** Biomechanical testing results after achilles repair. *p*-values comparing the two treatment groups are in bold.

	Experimental condition			*p*-values		
	AR (n = 12)	AR + OFS3 (n = 12)	Uninjured (n = 12)	**AR vs AR + OFS3**	Uninjured vs AR	Uninjured vs AR + OFS3
Failure Load N)	16.0 ± 2.9	17.4 ± 3.7	11.0 ± 1.5	**0.440**	<0.001	<0.001
Toughness (Nmm)	12.9 ± 4.3	16.8 ± 5.2	8.4 ± 2.1	**0.069**	0.027	<0.001
Stiffness (N/mm)	10.9 ± 2.3	12.0 ± 1.9	8.7 ± 1.8	**0.347**	0.036	0.001
Maximum displacement (mm)	1.9 ± 0.6	2.2 ± 0.6	1.9 ± 0.4	**0.335**	0.972	0.458

AR, achilles repair with saline; AR + OFS3 = Achilles repair with local OFS-3. Values given as mean ± standard deviation.

Compared to the uninjured hindlimbs used in pilot studies, both groups of repaired hindlimbs demonstrated significantly greater failure load, toughness, and stiffness. Displacement was similar between repaired and uninjured hindlimbs.

Biomechanical testing of 5–0 suture loops in isolation demonstrated significantly lower load to failure (13.1 N), decreased stiffness (1.2 N/mm), decreased toughness (76.8 Nmm), and increased maximum displacement before failure (12.2 mm) compared to the repaired hindlimb groups (*p* < 0.001 for all comparisons). Based on our force-displacement data for the suture, at a displacement of 2 mm, the sutures exhibited a mean tensile force of less than 0.4N, substantially lower than the failure loads observed in our tissue samples.

### 3.2 Histologic analysis

**FIGURE 3:**
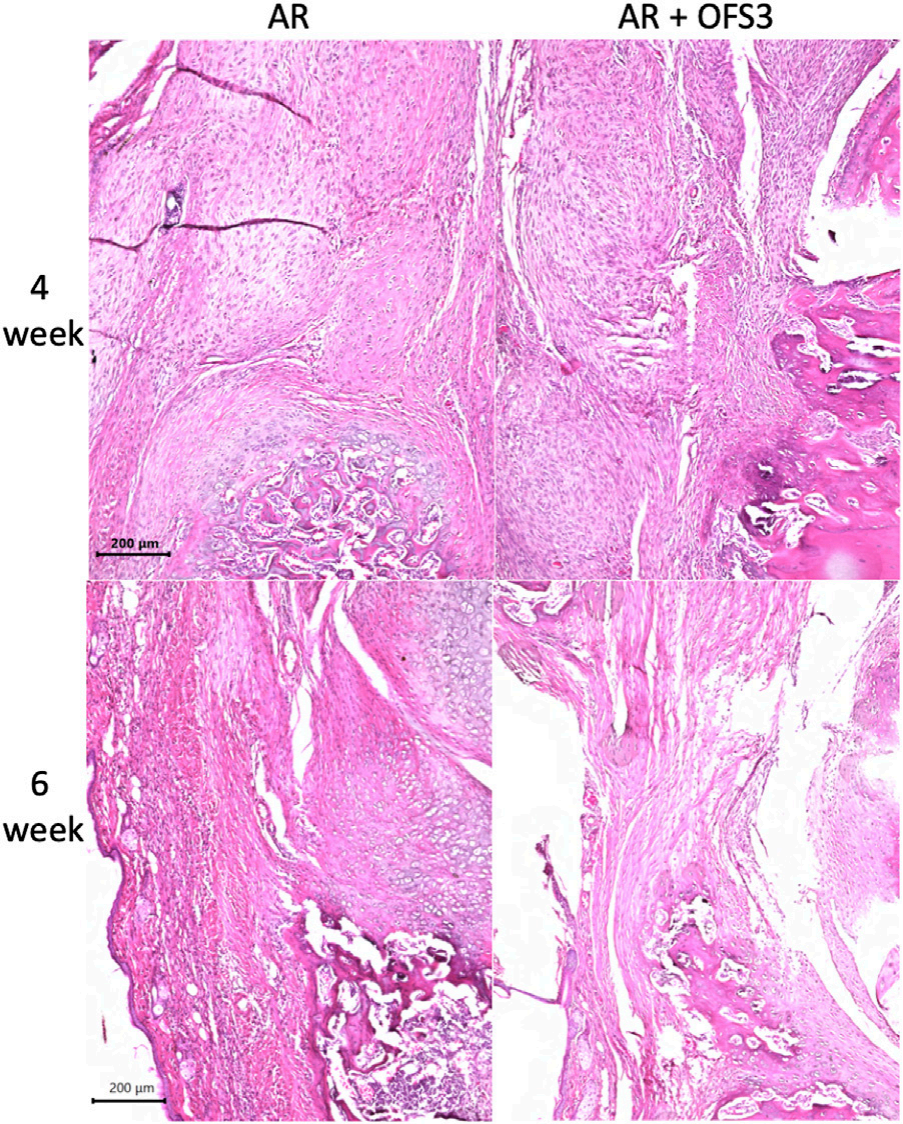
H + E results: Mouse hindfoot sagittal sections taken 4 and 6 weeks after Achilles tendon-to-bone repair followed by saline (AR) and Achilles tendon-to-bone repair followed by application of OFS-3 (AR + OFS-3), stained with H&E.

**FIGURE 4:**
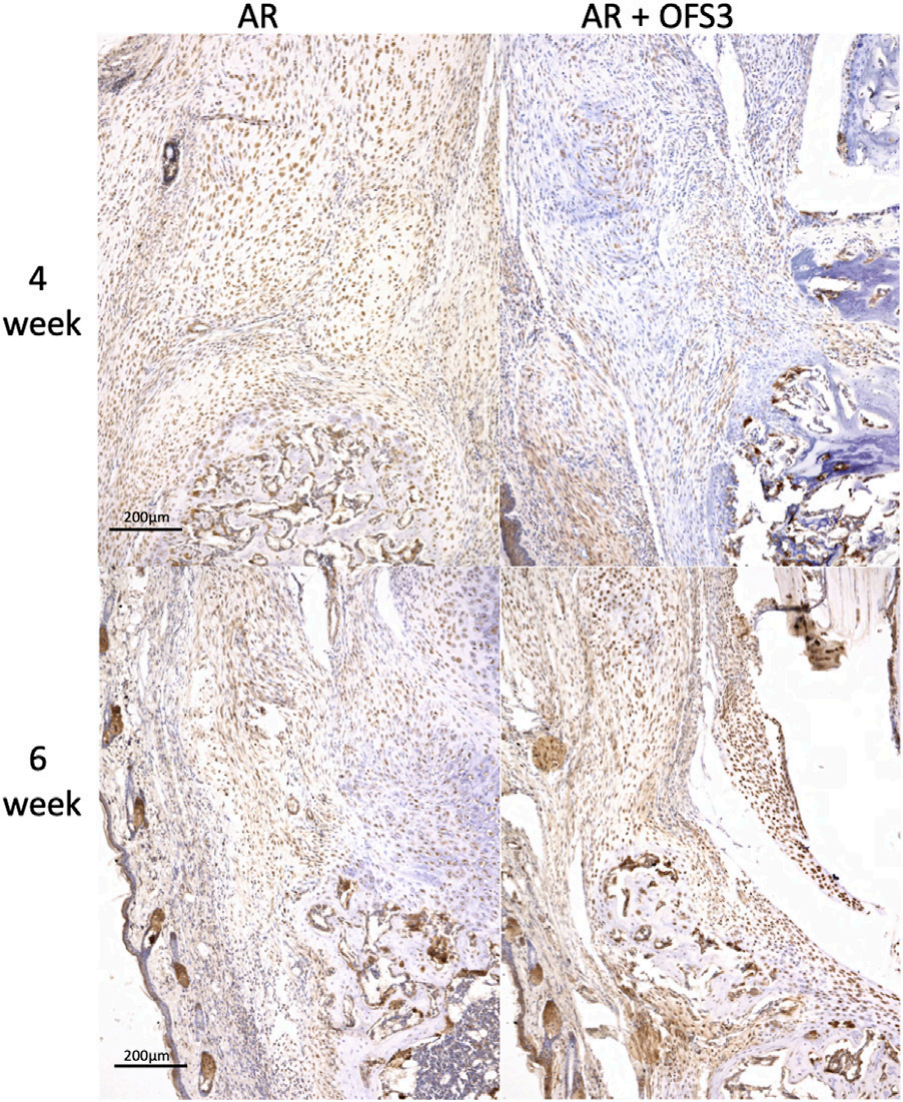
IHC results: Mouse hindfoot sagittal sections taken 4 and 6 weeks after Achilles tendon-to-bone repair followed by saline (AR) or OFS-3 (AR + OFS-3) treatment, stained with immunohistochemistry for Ctsk. Anti-Ctsk antibodies are stained brown while blue stain reflects residual hematoxylin.

**FIGURE 5:**
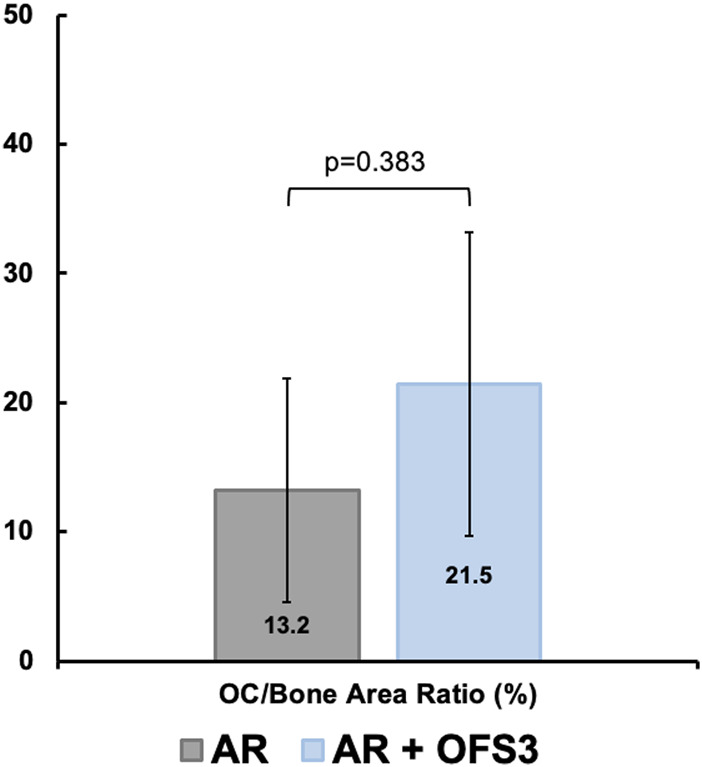
OC/total bone surface area ratio between AR and AR + OFS-3 groups.

Four weeks after surgery, animals in both groups (AR *versus* AR + OFS3) developed fibrotic tissue at the repair site with accumulation of fibroblast-appearing cells at the site of injury (Figure 3). Animals in both treatment groups had equivalent qualitative assessments of reparative tissue size and distributions of Ctsk-positive IHC staining throughout the calcaneus and Achilles stump, as noted by the distribution of brown DAB staining throughout each section (Figure 4). Of note, the reparative tissue at the repair site had a greater size in both the sagittal and coronal planes compared to the native tendon tissue at its insertion onto the calcaneus. Similar to our 4 weeks results, at 6 weeks after surgery, there was also no difference noted on qualitative assessments of the reparative tissue size, the fibrotic tissue appearance, or the intensity and distribution of Ctsk-positive IHC staining in animals that underwent Achilles repair followed by OFS-3 administration compared to saline control (Figure 4). Quantitative analysis demonstrated no statistically significant difference with regard to OC/bone area ratios between the AR and AR + OFS3 treatment groups (13.2% vs 21.5%, *p* = 0.3827) (Figure 5). For reference, the OC/bone area ratio in our uninjured specimens was approximately 6%.

**FIGURE 6:**
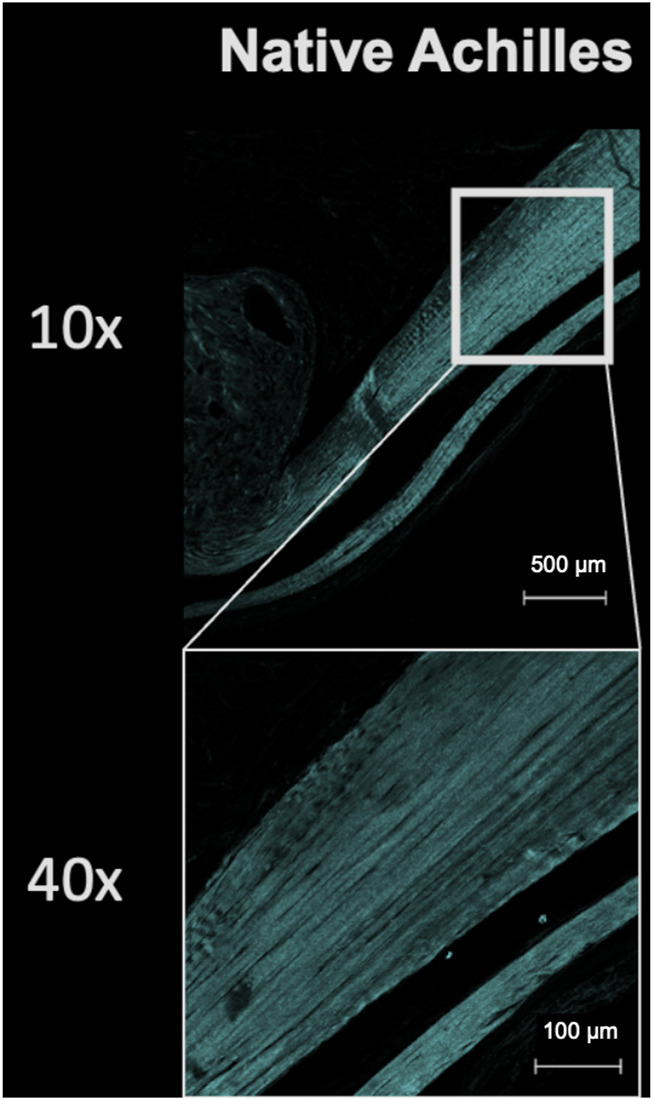
Second harmonic imaging of uninjured native Achilles. Collagen is displayed as cyan.

**FIGURE 7:**
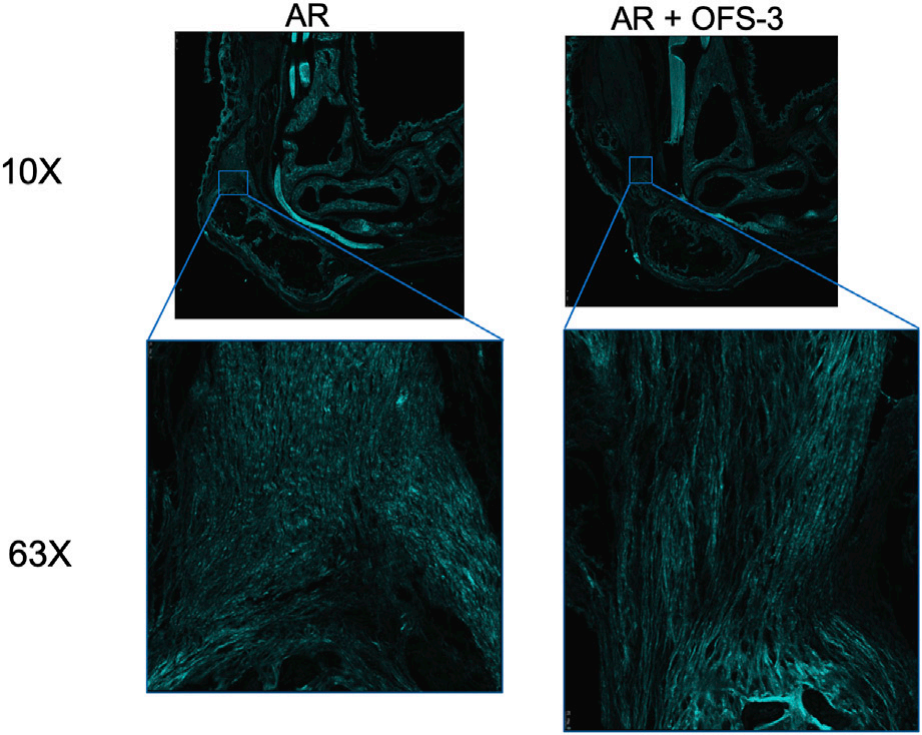
Second harmonic imaging of repaired specimens 6 weeks after Achilles tendon-to-bone repair followed by saline (AR) or OFS-3 (AR + OFS-3). Collagen is displayed as cyan.

**FIGURE 8:**
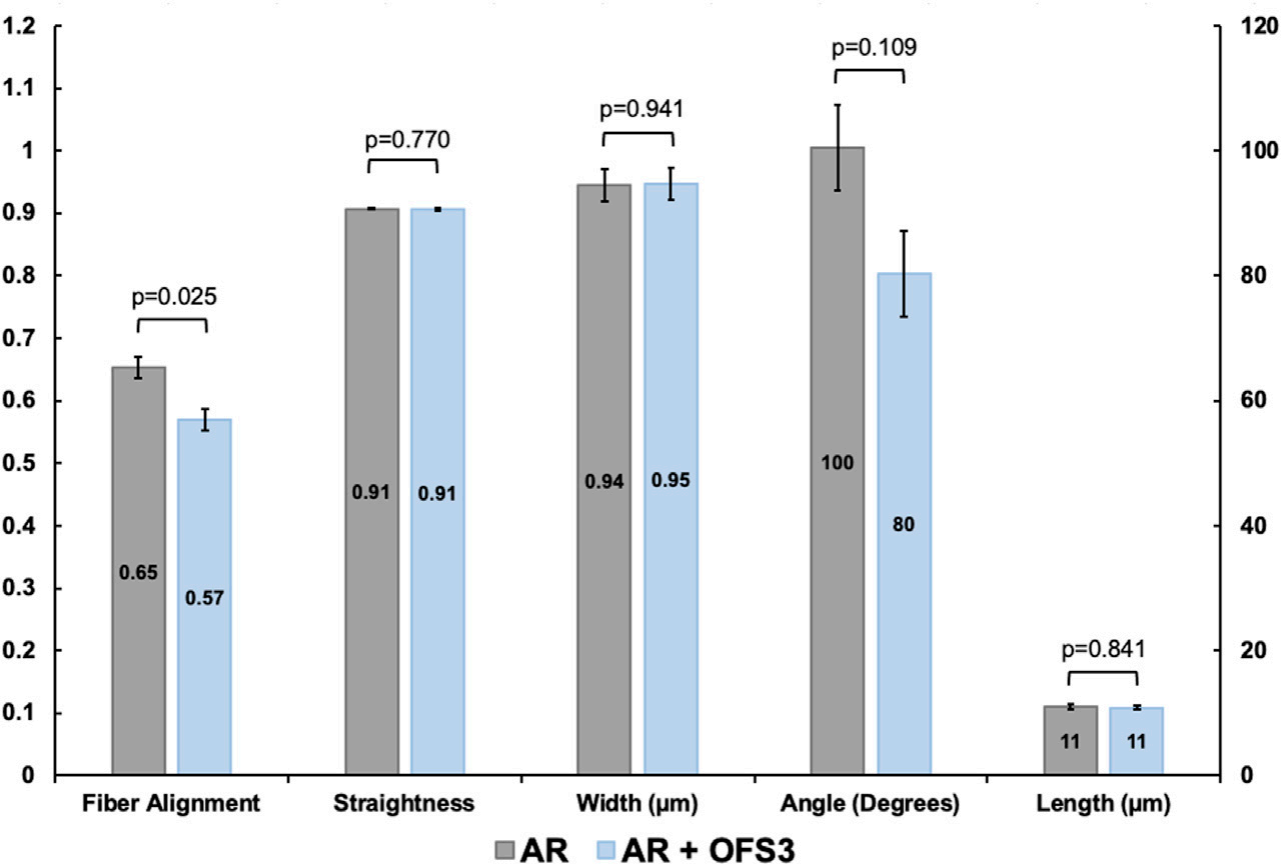
Collagen fiber dimensions and alignment between groups. Values for length and width are given in µm and angles in degrees. Straightness is unitless. For alignment, a value of 1 indicates all fibers are aligned in one direction and a value of 0 indicates fibers are not aligned.

Second harmonic generation imaging demonstrated disorganization of collagen at the repair site 6 weeks after tendon repair compared to native tendon (Figure 6; Figure 7). Quantitive analysis with CT-FIRE and CurveAlign demonstrated no significant difference in collagen fiber orientation angle (*p* = 0.109), length (*p* = 0.841), straightness (*p* = 0.770), or width (*p* = 0.941) between treatment groups (Figure 8). Collagen fiber alignment analysis demonstrated a significant mean difference between the AR and AR + OFS3 groups (mean 0.653 in AR, mean 0.569 in AR + OFS3, *p* = 0.0247). However, analysis of the components of variance demonstrated that 94% of the variation was due to replicate variability (SD = 0.185) where fiber to fiber variance was much larger than the variation between different regions of the mouse tendon repair sites or between different mice in each treatment group (mean difference of 0.084 is smaller than the replicate variability standard deviation of 0.185). Thus, the statistically significant mean difference in fiber alignment may not be clinically significant and may not be due to OFS-3 treatment.

## 4 Discussion

In this study, we used a murine Achilles repair model to determine the feasibility of using OFS-3 to target the site of tendon-bone repair without impairing the strength of repair or the biology of healing. We found that the addition of OFS-3 to tendon-to-bone repairs did not significantly affect the histological appearance of tendon healing and did not impact the biomechanical properties of the repaired tissue.

Our previously published *in vivo* imaging results demonstrated that bisphosphonate-based compounds reliably target the site of tendon-bone repair, and that release of coupled moieties can be controlled through a Ctsk-sensitive mechanism dependent on local osteoclast present at the repair site (Kremen et al., 2023). Fluorescent signal at the repair site remained elevated for over 2 weeks and was equivalent between animals that received OFS-3 locally or parenterally, illustrating the possibility of targeted yet delayed administration of the therapeutic agent beyond the time of the surgical procedure (Kremen et al., 2023). In the current study, we additionally report that application of OFS-3 does not significantly affect the histologic appearance, collagen alignment, or biomechanical strength of tendon-to-bone repair. These findings suggest that this BP-targeted Ctsk-coupled delivery scheme has minimal negative biologic consequences, justifying further development of this platform as a potential method for delivering bioactive agents such as BMP-2 directly to the site of tendon-to-bone repair through percutaneous or intravenous injection at time points beyond the time of surgery.

While previous studies have explored the use of bisphosphonates in targeting bone for the treatment of cancer or inflammatory bone disease (Uludag and Yang, 2002; Cole et al., 2016; Farrell et al., 2018), studies investigating the role of bisphosphonates in soft tissue-to-bone healing have focused on the effects of osteoclast inhibition on repair strength. Cadet et al. found that zoledronate improved bone density in a rat supraspinatus tear model (Cadet et al., 2010), while Thomopoulos et al. found that alendronate both prevented bone loss and improved load to failure in canine flexor tendon repairs (Thomopoulos et al., 2007). Conversely, Hjorthaug et al. found that the addition of zoledronate to a murine Achilles repair model significantly impaired the ultimate failure load and stiffness of the repaired tendon at 3 and 6 weeks after surgery (Hjorthaug et al., 2018). One of the reasons for the disparate findings between the current study and prior literature may be the choice of bisphosphonate. While the class of nitrogen-containing BPs (e.g., alendronate, zoledronate, pamidronate) exhibit anti-resorptive abilities due to their inhibition of FPPS, other bisphosphonates can be engineered to have minimal to no FPPS inhibition (Sung et al., 2020) while still maintaining their affinity for hydroxyapatite minerals, as is the case with OFS-3 (Hokugo et al., 2019; Okawa et al., 2022). Furthermore, BPs conjugated to other molecules, like OFS-3, may demonstrate even further attenuated antiresorptive effects compared to BPs used in isolation (Sun et al., 2016).

The addition of OFS-3 was not found to affect the histologic appearance, collagen organization, or progression of healing after Achilles tendon transection and repair. In both groups, a thick fibrotic callus developed at the site of the tendon transection by 4 weeks post-operatively, connecting the tendon stump to its bony footprint. At 6 weeks, the fibrous scar tissue demonstrated decreased cellularity and increased alignment of collagen fibers in the direction of stress, signifying unimpeded progression to the regenerative phase of tendon-to-bone healing in both groups (Bunker et al., 2014). At 6 weeks, we additionally found no significant difference in collagen angle, length, straightness, or width between animals that received OFS-3 or saline after Achilles repair. Finally, the distribution of Ctsk-positive IHC staining and the OC/bone area ratio was not significantly different between the two groups. The fact that treatment with OFS-3 after Achilles repair did not cause decreased OC surface area or decreased Ctsk levels (a proxy for osteoclast activity) suggests that OFS-3 does not significantly inhibit osteoclast activity. These findings are consistent to those reported by Hjorthaug et al. (Hjorthaug et al., 2018), who demonstrated that systemic administration of zoledronic acid in rats that underwent Achilles tendon-to-bone repair did not affect the size or organization of callus formation. Overall, while there remains a lack of consensus regarding the effect of clinically available osteoclast-inhibiting bisphosphonates on tendon-to-bone healing, our findings suggest that OFS-3, a molecule containing a BP with minimal, if any, FPPS inhibition, does not significantly impair tendon-to-bone healing.

Both groups of repaired hindlimbs demonstrated significantly higher failure load, toughness, and stiffness compared to the uninjured native hindlimbs among our study animals. As cross-sectional area is directly proportional to stiffness (K [stiffness] = E [Young’s Modulus] * Area/Length), the increased stiffness after repair is likely due to the increased size (area) and cellularity of the fibrotic reparative tissue. During dissection, it was noted that the fibrotic reparative tissue was notably larger in size (width and thickness) than native tendon tissue at its insertion onto the calcaneus. Silva et al. compared the biomechanical properties of native canine flexor digitorum tendons with those transected at their bony insertion, and found that the injured tendons demonstrated increased cellularity, thickness, and ultimate failure load at 21 days compared to native, uninjured tendons (Silva et al., 2004). Repaired tendons were also stiffer, exhibiting decreased displacement to failure compared to native tendons (Silva et al., 2004). Other animal studies have also reported that the scar tissue generated during the proliferative stage of Achilles tendon-to-bone healing results in increased failure load and stiffness relative to the native tendon insertion (Hibino et al., 2007; Hjorthaug et al., 2015). It is important to note that assessing stiffness parameters alone may not be the ideal metric for translating biomechanical findings to the *in vivo* condition. Shah et al. found repaired rat supraspinatus tendons to be thicker, yet weaker than native tendon tissue after cyclic loading and load-to-failure (Shah et al., 2017). Freedman et al. performed full thickness and partial (50%) width mid-substance Achilles transections in a murine model and found that although stiffness only decreased by 25%, the number of cycles to failure decreased by nearly 37-fold (Freedman et al., 2014). While their partial width tear model is expected to have different biomechanical findings to our tendon-bone repair model, Freedman’s findings demonstrate that small changes in stiffness may be associated with markedly different performance with cyclic loading. Thus, increased load to failure seen in repaired tendons may not translate to better performance with cyclic loading or when exposed to physiologic loading conditions.

Suture was not removed from repaired hindlimbs prior to our biomechanical testing due to two key reasons. First, clinically non-absorbable suture material is routinely implanted and maintained after surgical repair of tendons in humans. Thus, maintaining the suture in our specimens more accurate reflects a real-world protocol and could be considered more translationally relevant. Second, as noted above, healing tendon-bone repairs are associated with exuberant fibrotic reparative tissue (Silva et al., 2004). This reparative tissue often encases the suture material and attempting to remove the suture material risks damaging the tissue integrity at the repair site. To help determine the effect of retained sutures, we conducted an additional experiment to assess 5–0 nylon suture material in isolation and found that these suture loops had far lower load to failure and stiffness compared to the repaired tissue specimens. Since the hindlimb samples were around 10 times stiffer than the suture loops in isolation, each hindlimb specimen failed well before the maximum displacement of the suture loops. Therefore, the presence of the suture during mechanical testing is unlikely to have significantly affected our results, particularly in terms of the failure load of the healed tendon-bone repairs.

In summary, this study demonstrates that a bisphosphonate-based targeting and cathepsin K-coupled system can effectively deliver molecular cargo to the site of tendon-to-bone repair with minimal effect on the surrounding tissue in a mouse model of Achilles tendon-bone repair. Future studies are needed to evaluate whether this platform can effectively deliver bioactive agents such as BMP-2 or TGF-β.

### 4.1 Limitations

This study has several limitations. First, Achilles tendon transection is a sharp injury of healthy tissue, which may not accurately recapitulate tendon-bone repair in torn human diseased tendon tissue. Thus, the biomechanical findings in our murine Achilles tendon repair model among a relatively small number of male quadruped animals may not be generalizable to common clinical tendon injuries such as distal biceps tendon tears, pectoralis major tendon tears, or rotator cuff tendon tears. Second, the suture material at the repair site was not removed prior to biomechanical testing. Although we have discussed our rationale for this previously and our biomechanical testing of the suture material itself supports our rationale, this element of the study design can still be viewed as a limitation. Furthermore, although we noted that the reparative tissue at the site of tendon-bone repair was greater in size in both the sagittal and coronal planes, the cross-sectional area of this region was not quantified. As a result, normalization of force and displacement relative to cross-sectional area was not performed when evaluating the mechanical properties of our repaired hindlimbs. This is a limitation of this study. However, our primary aim was to study the mechanical behavior at the bone-tendon interface, an area characterized by the coexistence of heterogeneous tissue types and architecture with distinct mechanical properties. Given this heterogeneity, we believe that presenting unnormalized force and displacement data provides a more accurate representation of the mechanical behavior at this interface. Notably, this methodology is consistent with prior studies analyzing the bone-tendon interface which also did not normalize by cross-sectional area (Bell et al., 2015; Cong et al., 2018). This study also did not quantify the effect of OFS-3 treatment on local gene expression in the repaired tissue. While gene expression analysis of repaired tissue with and without OFS-3 administration may be informative and represents a potential area of future study, this study primarily aimed to assess whether the addition of OFS-3 would lead to worse tendon-bone healing strength and altered biomechanical properties. In addition, only one dose of this BP-based molecule delivered at one time point was evaluated in this preliminary study. Different doses and additional administrations may affect the histologic and biomechanical testing results. Also noteworthy, our findings are specific to OFS-3, a novel modified pamidronate-based BP molecule. Different bisphosphonates can have significantly different degrees of osteoclast inhibition (Hokugo et al., 2019; Okawa et al., 2022), which may have alternative effects on outcome measures. Finally, this investigation is an early-phase translational investigation of a method for targeting molecules to the site of tendon-bone repair. The efficacy of this approach as a growth factor delivery strategy that may enhance soft tissue-to-bone healing has not yet been demonstrated.

### 4.2 Conclusion

OFS-3 did not significantly affect the biomechanical properties or histologic appearance of murine Achilles tendon-to-bone repairs. This study shows that a BP-based Ctsk-coupled target-and-release drug delivery strategy can be executed in a manner that does not affect the biomechanical integrity or histologic organization of tendon-to-bone repairs.

## Data Availability

The raw data supporting the conclusions of this article will be made available by the authors, without undue reservation.
